# A Short Peptide That Mimics the Binding Domain of TGF-β1 Presents Potent Anti-Inflammatory Activity

**DOI:** 10.1371/journal.pone.0136116

**Published:** 2015-08-27

**Authors:** Emília R. Vaz, Patrícia T. Fujimura, Galber R. Araujo, Carlos A. T. da Silva, Rangel L. Silva, Thiago M. Cunha, Mônica Lopes-Ferreira, Carla Lima, Márcio J. Ferreira, Jair P. Cunha-Junior, Ernesto A. Taketomi, Luiz R. Goulart, Carlos Ueira-Vieira

**Affiliations:** 1 Laboratory of Nanobiotechnology Institute of Genetics and Biochemistry, Federal University of Uberlândia, Uberlândia, Minas Gerais, Brazil; 2 Department of Pharmacology, School of Medicine of Ribeirão Preto, University of São Paulo, Ribeirão Preto, São Paulo, Brazil; 3 Immunoregulation Unit, Special Laboratory of Applied Toxicology (CEPID/FAPESP), Butantan Institute, São Paulo, São Paulo State, Brazil; 4 Laboratory of Immunotechnology and Immunochemistry, Institute of Biomedical Sciences, Federal University of Uberlândia, Uberlândia, Minas Gerais, Brazil; 5 Department of Medical Microbiology and Immunology, University of California Davis, Davis, CA, United States of America; French National Centre for Scientific Research, FRANCE

## Abstract

The transforming growth factor beta 1 (TGF-β1) is a pleiotropic cytokine with multiple roles in development, wound healing, and immune regulation. TGF-β1-mediated immune dysfunction may lead to pathological conditions, such as inflammation. Chronic inflammatory process is characterized by a continuous release of pro-inflammatory cytokines, and the inhibition or the blockage of these cytokines signaling pathways are considered a target treatment. In this context, despite the high numbers of TGF-β-targeted pathways, the inducible regulatory T cells (iTreg) to control inflammation seems to be a promising approach. Our aim was to develop novel peptides through phage display (PhD) technology that could mimic TGF-β1 function with higher potency. Specific mimetic peptides were obtained through a PhD subtraction strategy from whole cell binding using TGF-β1 recombinant as a competitor during elution step. We have selected a peptide that seems to play an important role on cellular differentiation and modulation of TNF-α and IL-10 cytokines. The synthetic pm26TGF-β1 peptide tested in PBMC significantly down-modulated TNF-α and up-regulated IL-10 responses, leading to regulatory T cells (Treg) phenotype differentiation. Furthermore, the synthetic peptide was able to decrease leukocytes rolling in BALB/C mice and neutrophils migration during inflammatory process in C57BL/6 mice. These data suggest that this peptide may be useful for the treatment of inflammatory diseases, especially because it displays potent anti-inflammatory properties and do not exhibit neutrophils’ chemoattraction.

## Introduction

The inappropriate response of the immune system caused by pathogens and self-antigens results in a pathological inflammation that induces tissue destruction [1]. T cells activation that survived to the induction of central or peripheral tolerance have the potential to develop a chronic inflammation and autoimmune diseases, such as rheumatoid arthritis and diabetes type I [2]. Pro-inflammatory cytokines, such as tumor necrosis factor alpha (TNF-α), are key features of inflammatory process and may cause tissue damage and destruction [3]. This cytokine acts in vasodilation, edema, leukocyte adhesion to epithelium, macrophage activation, fever, metalloproteases activation, and contributes to the oxidative stress at inflammation sites [4]. TNF-α signaling pathway defects, including their receptors and the nuclear factor-kappaB (NFκB) activation, can be found in human immune disorders and murine disease models [5, 6]. Furthermore, TNF-α inhibits the anti-inflammatory cytokine Interleukin-10 (IL-10) production [7], triggering chronic inflammation [8] and autoimmune diseases [9]. In patients with rheumatoid arthritis, TNF-α is found in the synovial liquid contributing to joint inflammation [10]. The modulation of these cytokines is essential to stabilize the immune response, leading to reduced inflammation and restoring the self-tolerance [11]. Mouse models have greatly contributed to our understanding of several human immunological mechanisms. In this context, BALB/C and C57BL/6J mice have been widely used in different studies in many different research areas [12–14].

Modulation of regulatory T cells (Tregs) is a powerful tool to induce immune tolerance [15], and may be a strategy to control inflammatory and autoimmune responses [16],[17]. Thymus-derived cells (nTreg) and those induced in the periphery (iTreg) share the same cell markers (CD4^+^CD25^+^Foxp3^+^). In general, iTregs can be peripherally induced by TGF-β1 stimulation [17], releasing immunomodulatory cytokines such as IL-10 and the transforming growth factor beta 1 (TGF-β1) [18]. A major advantage of iTreg in comparison to nTreg is that it is more stable and effective in treatments involving inflammatory processes and autoimmune diseases [19],[20]. Thus, the Treg modulation may be considered a promising tool for inflammatory diseases treatment [21].

TGF-β1 is a pleiotropic cytokine [22] and its signaling activation has been proposed as a control mechanism of reactive peripheral T cells [23]. TGF-β1 binds to TβRII receptor and promotes phosphorylation of TβRI, and SMAD 2 and 3 proteins. SMADS are phosphorylated on two serine residues in the carboxy-terminal (C-terminal), enabling a complex formation with SMAD 4, which interacts with co-activators and co-repressors, translocate into the nucleus and regulate the transcription of target genes, including FOXP3, by direct binding to DNA [24]. Another form of signaling is through SMAD independent pathways, for example the protein kinases GTPase, phosphatidylinositol 3 kinase [25],[26], p38 TRAF6-TAK1 and c-Jun NH(2)-terminal kinase (JNK) that are also induced by TGF-β1, whereas it down-regulates the mitogen-activated protein kinases (MAPK) cascade [26],[27] and NFκB transcription [28].

The growing interest in the TGF-β1 pathway as a treatment option for inflammatory diseases, especially due to the possibility of controlling reactive T cells, led us to the development of novel TGF-β1-like peptides that could outperform the effects of TGF-β1. One of the mimetic peptides selected played an important role on cellular differentiation and modulation of TNF-α and IL-10 responses, key cytokines involved in inflammation, which will be discussed herein.

## Materials and Methods

### Blood samples and animals

Peripheral blood mononuclear cells (PBMC) were obtained from twenty healthy volunteers using Ficoll-Paque PLUS (GE Healthcare) following to manufactures protocol. The Ethics Committee on Human Research at the Federal University of Uberlândia (CEP 449/10) approved all procedures and all subjects enrolled in this study signed informed consent form. A total of thirty-five C57BL/6 mice and six BALB/C mice were housed at the animal facility of the Federal University of Uberlândia where they fed *ad libitum*. The mice were housed in a conventional animal house with 12-h dark/light cycles. The Ethics Committee on Animal Research at the Federal University of Uberlândia (CEUA/UFU 020/12) approved all procedures. This study was carried out in strict accordance with the recommendations contained in terms for the use of animals in research and teaching of the Federal University of Uberlândia (Uberlândia, MG, Brazil), in compliance with the National Guidelines, as set forth by the Institutional Animal Care (Law number 11.794, 2008). All efforts were made to minimize mice suffering.

### Phage Display

To select peptides with binding affinity to TGF-β1 receptors, a PhD-7mer phage display peptide library Kit (New England Biolabs) was used. This is a combinatorial library containing billions of pooled peptides presented on phage particles, where 10^10^ different peptides can be screened simultaneously for the desired activity [29]. In order to select peptides that could mimic TGF-β1, a total of 1x10^6^ PBMC were incubated with the PhD-7mer library containing 1 × 10^11^ phage particles under gentle agitation for 1 hour at 4°C (to avoid endocytosis). The unbound phages were discarded by washing five times with PBS (137 mM NaCl, 10 mM phosphate, 2.7 mM KCl, and pH 7.4). Phages that were bound to receptors onto cells surface were competitively eluted with 10 ng/mL of recombinant TGF-β1 (Sigma-Aldrich). The eluted phages were amplified in *E*. *coli* ER2738 strain (New England Biolabs), purified by PEG-8000/NaCl precipitation and used in the next round of selection. Three rounds of selection were performed for the enrichment of specific peptides. Titration was performed after each of the three rounds of selection to evaluate the rate of recovered phage clones.

### DNA sequencing

A total of forty-six phage clones were submitted for sequencing. For DNA extraction, an overnight culture of phages was suspended in a 100 μL of sodium-iodide buffer (10 mmol/L Tris-HCl, pH 8.0, 1 mmol/L EDTA, 4 mol/L NaI) followed by precipitation with absolute ethanol. Phage DNA was centrifuged at 10,000 rpm for 10 minutes, washed with 70% ethanol, and resuspended in 30 μL of ddH_2_O. Phage DNA quality was verified in 0.8% agarose gel electrophoresis prior to sequencing. For sequencing, a total of 50 ng of DNA was mixed with -96 M13 sequencing primer (5’-OH CCC TCA TAG TTA GCG TAA CG-3’, Biolabs) and the sequencing mix (DYEnamic ET Dye Terminator Cycle Sequencing Kit, Amersham Biosciences). Sequences detection was performed in a MegaBace 1000 Genetic Analyzer (Amersham Biosciences) automatic capillary sequencer.

### Phage-ELISA

Nearly 1x10^6^ PBMC diluted in bicarbonate buffer (0.1 M NaHCO_3_ and 0.1 M Na_2_CO_3_, pH 9.4) were coated in a 96-well Maxisorp microtiter plate (Nunc, Denmark) and incubated overnight at 4°C. After blocking with PBS-BSA 5% (Sigma-Aldrich) at 37°C for 1 hour, each well was washed once with PBS, and 1x10^11^ selected phages were added into each well, which was further incubated at 37°C for 1 hour. Each well was washed five times with PBS. Anti-M13 antibody labeled with peroxidase (Amersham Biosciences) diluted at 1:5000 was added into each well followed by incubation for 1h at 4°C. Each well was washed five times, revealed with OPD SigmaFast (Sigma-Aldrich), stopped by 4N H_2_SO_4_ and read at 492 nm in a microplate reader (Titertek Multiskan Plus, Flow Laboratories, USA).

### Peptide synthesis

The pm26TGF-β1 peptide was chemically synthesized by GenScript USA Inc (Piscataway, NJ, USA). The peptide was constructed with 14 residues (ACESPLKRQCGGGS) following phage display manual [30].

### *In silico* analysis

DNA sequences were translated by ExPASy Translate tool (http://web.expasy.org/translate/). The Pepitope Server (http://pepitope.tau.ac.il/index.html) was used for three-dimensional analysis by predicting the alignment of the peptides with the TGF-β1 structure (PDB: 1KLC). Structural predictions were performed by the Raptorx program (http://raptorx.uchicago.edu/). Peptide interactions with TGF-β1 receptor (PDB: 1PLO) were evaluated by the Pathdock program (http://bioinfo3d.cs.tau.ac.il/PatchDock/).

### Cytotoxicity assay

PBMC from healthy donors were obtained after Ficoll density gradient centrifugation from heparinized blood samples. Nearly 1x10^5^ PBMC were maintained in RPMI-1640 medium (Gibco), supplemented with 10% fetal bovine serum and 1% gentamicin (Sigma-Aldrich) (complete medium) under standard culture conditions (37°C, 95% humidified air, and 5% CO2). Cells were treated with the pm26TGF-β1 synthetic peptide at 1 μM, 10 μM and 100 μM for 24 h. Then, 10 μL of 3-(4,5-dimethylthiazol-2-yl)-2,5-diphenyltetrazolium bromide (MTT) (Calbiochem, Darmstadt, Germany) solution (5 mg/ml) were added to each well, and the culture was further incubated for 4 h at 37°C. A total of 50 μL of N-dimethylmethanamide solution were added to each well followed by overnight incubation. The absorbance of each well was determined on a microplate reader at 592 nm. The relative cell viability (%) was calculated using the formula: % Viability = [(A_592 –_treated cells)/(A_592_ –untreated cells)] x 100. Negative control cells were treated with RPMI.

### Cellular Stimulations and cytokine levels

Nearly 1x10^6^ PBMC were treated with the pm26TGF-β1 synthetic peptide at 1 μM, 10 μM and 100 μM for 1 hour, followed by incubation with Lipopolysaccharides (LPS) (Sigma-Aldrich), Phorbol-12-myristate-13-acetate (PMA) (Sigma-Aldrich) or the recombinant TGF-β1 (Sigma-Aldrich) for 24 or 48 hours. After stimuli, pro- (TNF-α) and anti- (IL-10) inflammatory cytokines were quantified by commercial ELISA kit, according to the manufacturer’s instructions (eBiosciences). All assays were performed in triplicates.

### Flow Cytometry

PBMC were isolated and stimulated for 1 hour with the pm26TGF-β1 synthetic peptide at 1 μM, 10 μM and 100 μM. After peptide stimuli, the recombinant TGF-β1 (10 ng/mL) (Sigma-Aldrich) or PMA (50 ngm/mL) was added and further stimulated for 24 hours followed by Tregs percentage analysis. Cell labeling was performed according to the manufacturer's instructions (BD Biosciences). The antibodies anti-CD4 (APC) and anti-CD25 (PE-Cy7) were used for surface receptors labeling, and anti-Foxp3 (FITC) for intracellular labeling. Cells were analyzed on a BD FACSAria III flow cytometry (BD, Biosciences). Lymphocytes were first gated according to their size and granularity. Thus, the gate was placed on the subpopulation that represented viable CD4 cells, and analysis was focused on cell subsets that were CD25^+^ and Foxp3^+^.

### Intravital Microscopy

The dynamics of alterations in the microcirculatory network were determined using intravital microscopy (IVM) as described elsewhere [31]. Briefly, BALB/C mice, male, were anesthetized by an intraperitoneal (i.p.) injection containing 2% Xylasine (Calmiun) and 0.5 g/Kg of ketamine (Holliday-Scott SA). The animals were kept on a platform thermally controlled at 37°C and then the scrotum was opened and cremaster muscle exteriorized. After longitudinal incision with a cautery and spreading of the muscle over a cover glass, the epididymis and testis were mobilized and pinned aside leading to full microscopic access to the cremaster muscle microcirculation. Then, exposed tissue was applied topically with 20 μl of warmed PBS (pH 7.4), LPS (0.02 μg/mL) (*E*. *coli* 055:B5; Sigma-Aldrich) or pm26TGF-β1 synthetic peptide at 1 μM after LPS application. The postcapillary venules, with a diameter of 25–40 μm, were chosen and the mean rolling leukocyte flux was determined 30 minutes after application by counting the number of cells passing a fixed line perpendicular to the axis of each vessel over a period of 1 minute. This microvascular study by transillumination of tissue was completed with the aid of the optical microscope (Imager A1, Carl-Zeiss, Oberkochen, DE) coupled to a camera (AxionCam, ICc1) using objective opening/longitudinal distance ×10/0.3 and optovar 1.6x.

### Peritonitis Assay

Peritonitis assay was performed as described elsewhere [14]. Briefly, C57BL/6 mice, male, 5 to 7-weeks old with 20 to 30g of weight, were subcutaneously injected with vehicle solution (PBS), pm26TGF-β1 synthetic peptide at 100 mg, 300 mg or 1000 mg/kg, or dexamethasone (2 mg/kg) (Sigma-Aldrich). Seven mice composed each group. After 1-hour pretreatment, mice were injected i.p. with 500 μL carrageenan (500 μg/cavity), and after 4 hours, animals were euthanized and migrant leukocytes were extracted by washing the peritoneal cavity with 1.5 mL of PBS/EDTA (0.372 mg / mL). Total cell count was performed in a cell counter (Coulter T AC; Coulter Corporation, Miami, FL, USA). Differential cell counts were performed in samples fixed on slides by cytospin (Shandon Lipshaw Inc.), followed by hematoxylin and eosin staining, and analysis was carried out in a white-light microscope. The percentage of neutrophils was defined by the proportion of differential counting over the total cell count to establish the number of migrant neutrophils.

### Pm26TGF-β1 detection by ELISA

Nearly 1x106 PBMC diluted in bicarbonate buffer (0.1 M NaHCO3 and 0.1 M Na2CO3, pH 9.4) were coated in a 96-well Maxisorp microtiter plate (Nunc, Denmark) and incubated overnight at 4°C. After blocking with PBS-BSA 5% (Sigma-Aldrich) at 37°C for 1 hour, each well was washed once with PBS. Pm26TGF-β1 (1 μg/mL) or TGF-β1 (8000pg/mL) was added and then the plate was incubated at 37°C for 1 hour. Each well was washed five times with PBS. Anti-TGF-β1 antibody labeled with HRP (BD Bioscience) diluted at 1:250 was added into each well followed by incubation for 1h at 4°C. Each well was washed five times, revealed with OPD SigmaFast (Sigma-Aldrich), stopped by 4N H2SO4 and read at 492 nm in a microplate reader (Titertek Multiskan Plus).

### Statistical analyses

Bonferroni test was used to analyze the differences between groups in the *in vivo* experiments. Tukey test was used to analyze the statistical significance of decreased leukocyte migration. Kruskal-wallis test was used to analyze the Treg percentage after treatments. *P* values less than 0.05 were considered statistically significant.

## Results

### Peptides selection

We have performed phage display against whole cell surface of PBMCs without disturbing the cell integrity instead of using the extracellular domain of the recombinant human TβRII, because we hypothesized that the 3D interaction between TGF-β1 and its receptor is a critical event to the affinity of both, thus reactive peptides originated from the phage library may behave like TGF-β1 with the same conformational interaction. Therefore, to further improve affinity during peptides selection, we have performed a competitive elution with recombinant TGF-β1 in each cycle. A total of 48 phage clones were obtained after three rounds of selection, where 13 presented different peptides (Table 1).

**Table 1:** Sequence and frequency of the selected peptides.

Peptide	Sequence	Frequency (%)
pm1TGF-β1	FLPASGL	1/48 (2,08)
pm2TGF-β1	PWPLPYL	1/48 (2,08)
pm3TGF-β1	WGLLDLT	1/48 (2,08)
pm5TGF-β1	PAERLRS	2/48 (4,16)
pm7TGF-β1	RNLDGWS	1/48 (2,08)
pm10TGF-β1	NLSSSWI	3/48 (6,25)
pm11TGF-β1	TLPSNTH	1/48 (2,08)
pm12TGF-β1	MSAFPFL	1/48 (2,08)
pm13TGF-β1	SRLGQYI	1/48 (2,08)
pm14TGF-β1	PFGPLPP	1/48 (2,08)
pm18TGF-β1	TIASTLH	1/48 (2,08)
pm19TGF-β1	PRAPADV	1/48 (2,08)
pm26TGF-β1	ESPLKRQ	1/48 (2,08)

**Fig 1:**
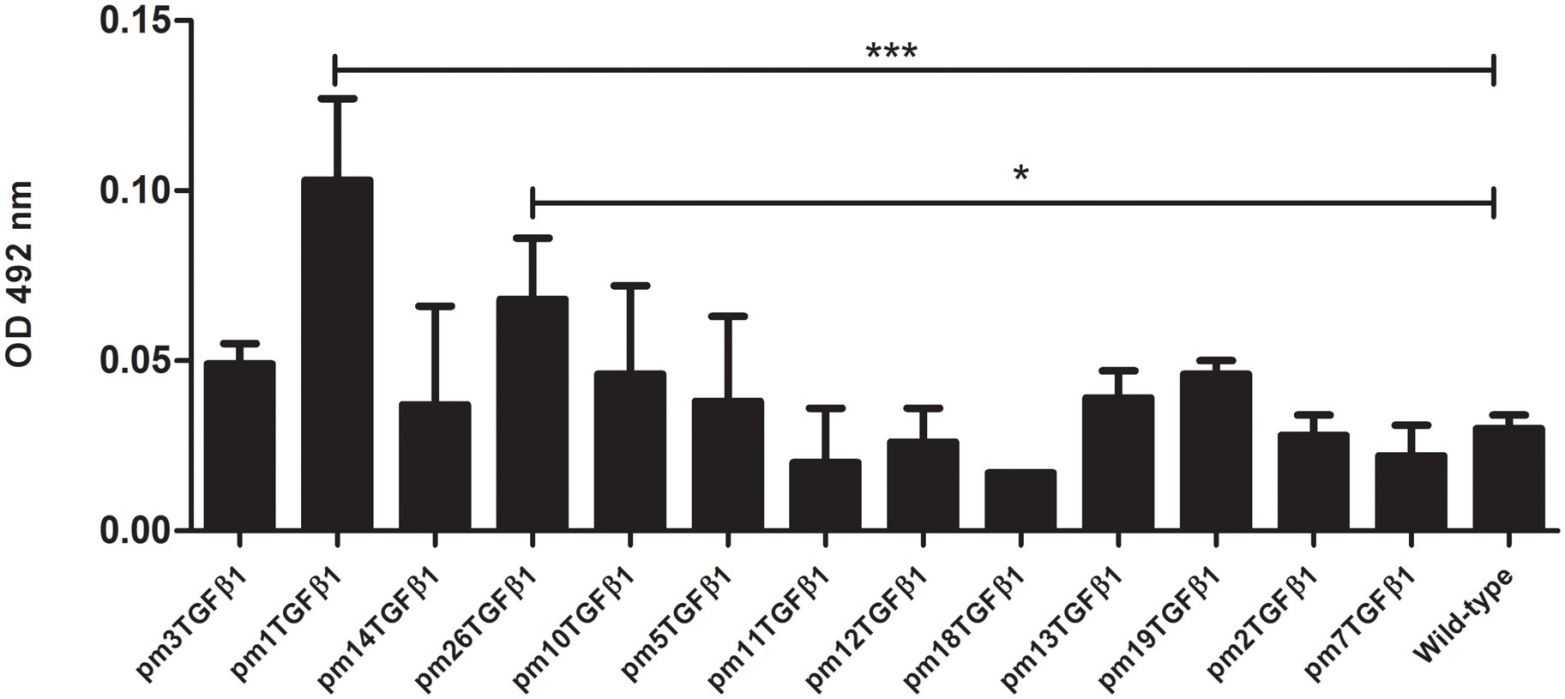
Phage-ELISA showing the reactivity obtained through the interaction of the selected phage clones and PBMC obtained from healthy donors. The pm1TGF-β1 and pm26TGF-β1 phage clones showed significant reactivity (P < 0.0001 and P < 0.05, respectively) compared to wild-Type phage.

To further demonstrate the reactivity of the selected clones to PBMCs, phage-ELISA assay was carried out (Fig 1). The wild-type phage (displaying no peptide on its surface) used as a negative control. The binding intensity of both pm1TGF-β1 and pm26TGF-β1 phage clones was significantly higher than those obtained from the other selected clones and the negative control (P < 0.05).

### Bioinformatics analysis

**Fig 2:**
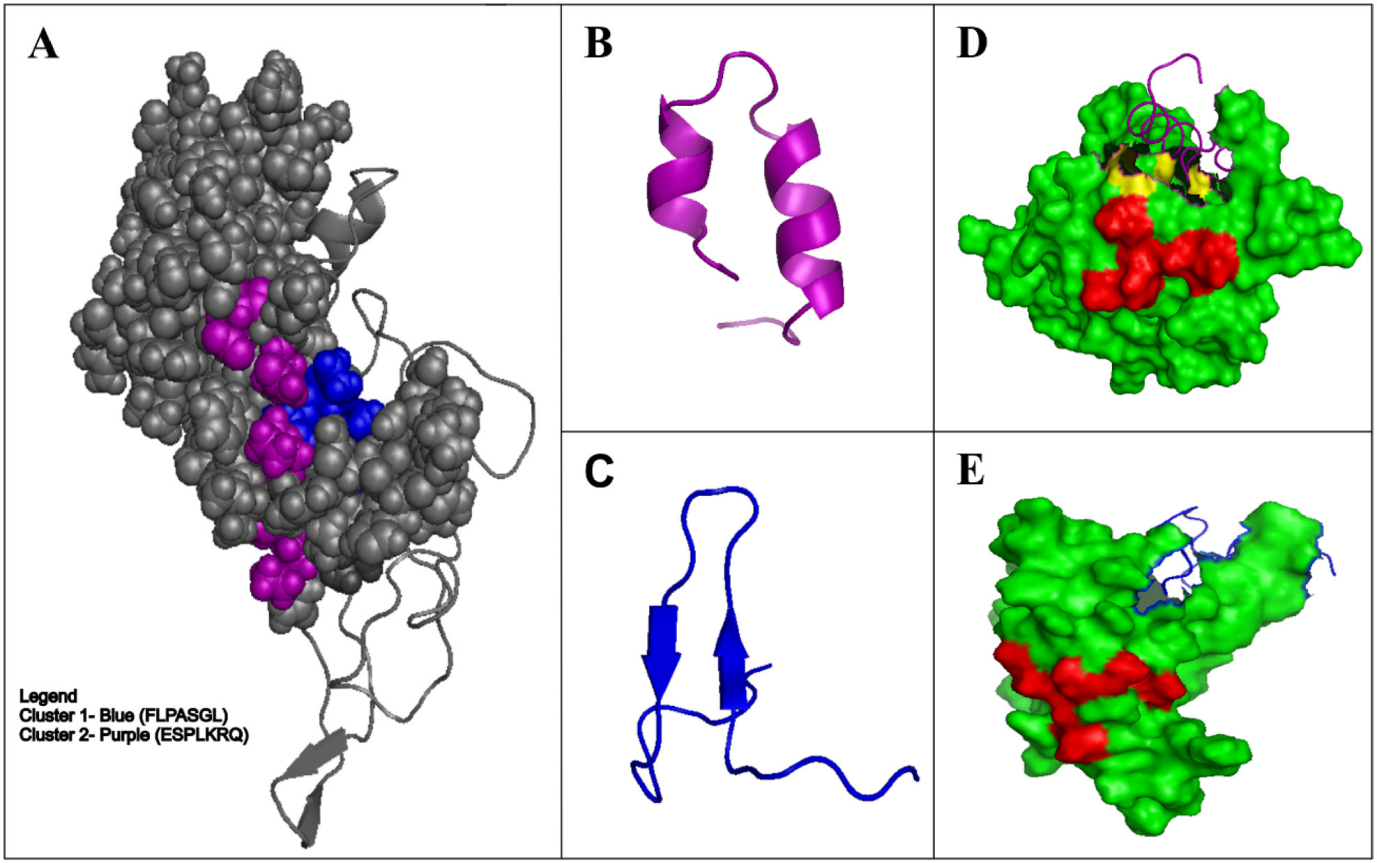
Three-dimensional analysis showing similarities between the pm1TGF-β1 and pm26TGF-β1 peptides and the TGF-β1 molecule. (A) Regions of similarity between the pm1TGF-β1 peptide and the TGF-β1 molecule are shown in blue while regions of similarity between the pm26TGF-β1 peptide and the TGF-β1 molecule are shown in purple. (B) Pm26TGF-β1 peptide α-helix structure. (C) Pm1TGF-β1 peptide β-sheet-like structure. (D) The region of interaction between the pm26TGF-β1 peptide (purple) and TβRII (green). The region of interaction between the TGF-β1 molecule and the TβRII is represented in red. The binding site shared by TGF-β1 and pm26TGF-β1 peptide is represented in yellow. (E) The region of interaction between the pm1TGF-β1 peptide (blue) and TβRII (green). The region of interaction between the TGF-β1 molecule and the TβRII is represented in red.

**Fig 3:**
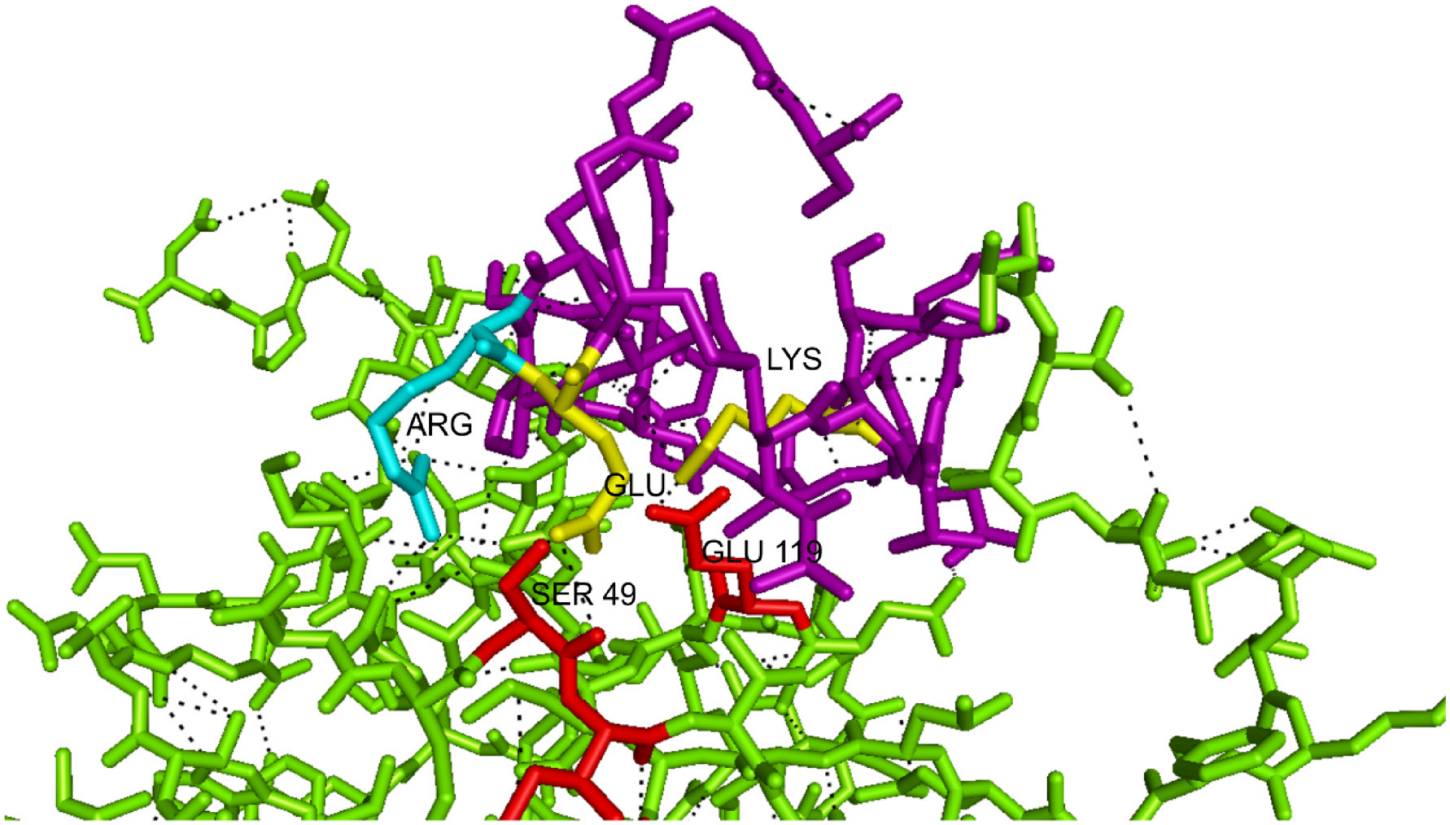
Three-dimensional analysis showing the region of interaction between the pm26TGF-β1 peptide and TβRII. The interaction between the Glu and Lys residues present in the pm26TGF-β1 peptide (purple) and the Ser^49^ Glu^119^ residues of the TβRII (red). The binding site shared by TGF-β1 and pm26TGF-β1 peptide is represented in yellow. The Arg residue present in the pm26TGF-β1 peptide is represented in cyan.

In order to verify whether the two most reactive peptides (pm1TGF-β1 and pm26TGF-β1) were similar to TGF-β1, we have performed alignment with the three-dimensional structure of TGF-β1. Interestingly they were located in two different regions of the cytokine (Fig 2A). The predicted three-dimensional structure analysis of the two peptides also showed different conformations between them, in which the pm26TGF-β1 presented an α-helix type structure (Fig 2B), while the pm1TGF-β1 presented a β-sheet-like structure (Fig 2C). Their predicted interactions with TβRII are shown in Fig 2D and 2E. The Glu and Lys in pm26TGF-β1 interacts with the Ser^49^ and Glu^119^ of the TβRII activation site. The pm26TGF-β1 peptide also has Arg residue, which interacts with the TβRII by two hydrogen bounds (Fig 3). The pm1TGF-β1 peptide did not present any residue interacting in the same region, and for this reason, the pm26TGF-β1 peptide was chosen for further investigations.

### The pm26TGF-β1 peptide is non-cytotoxic and mediates an anti-inflammatory response

**Fig 4:**
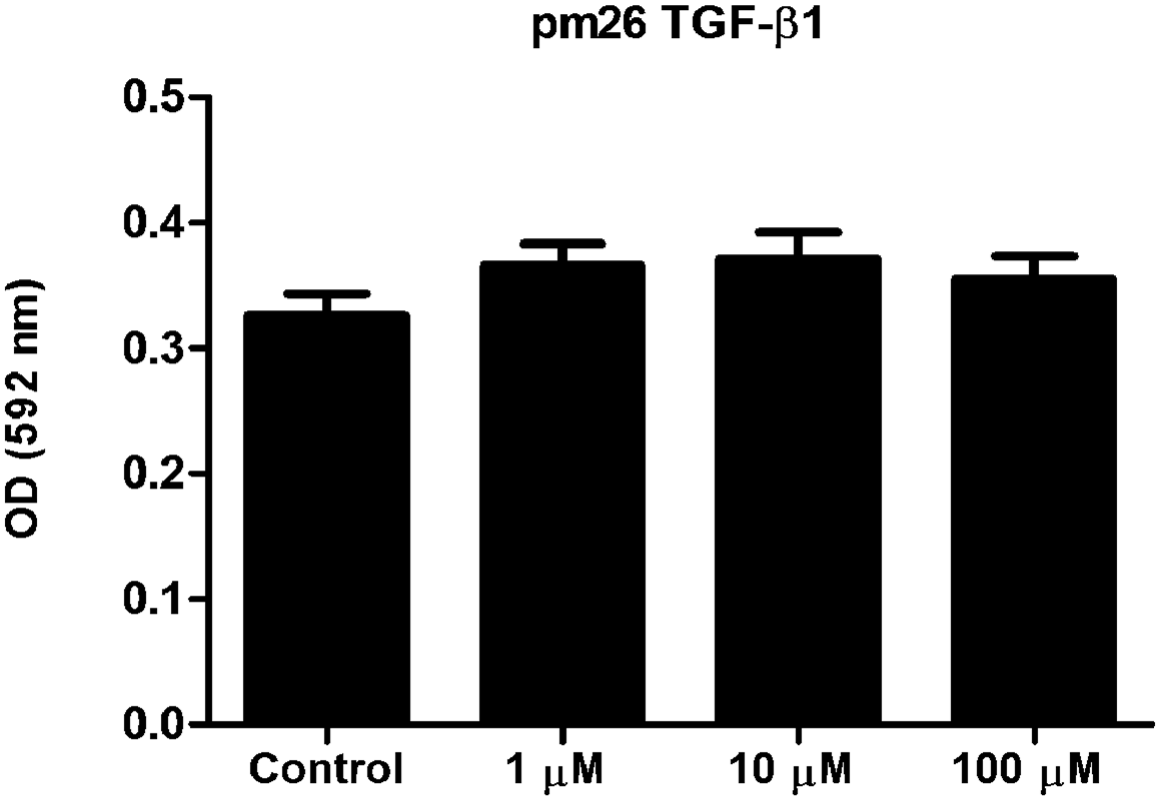
Cytotoxicity analysis of the synthetic peptide in PBMC. The pm26TGF-β1 peptide showed no statistical difference when compared to control (PBMC without treatment) indicating that the tested concentrations did not have a cytotoxic effect.

To demonstrate that the pm26TGF-β1 peptide mimics the TGF-β1 without cytotoxic action, we performed the MTT assays using PBMC. It was shown that the pm26TGF-β1 peptide tested at 1 μM, 10 μM and 100 μM concentrations did not affect cells viability and presented no significant differences from controls (Fig 4).

**Fig 5:**
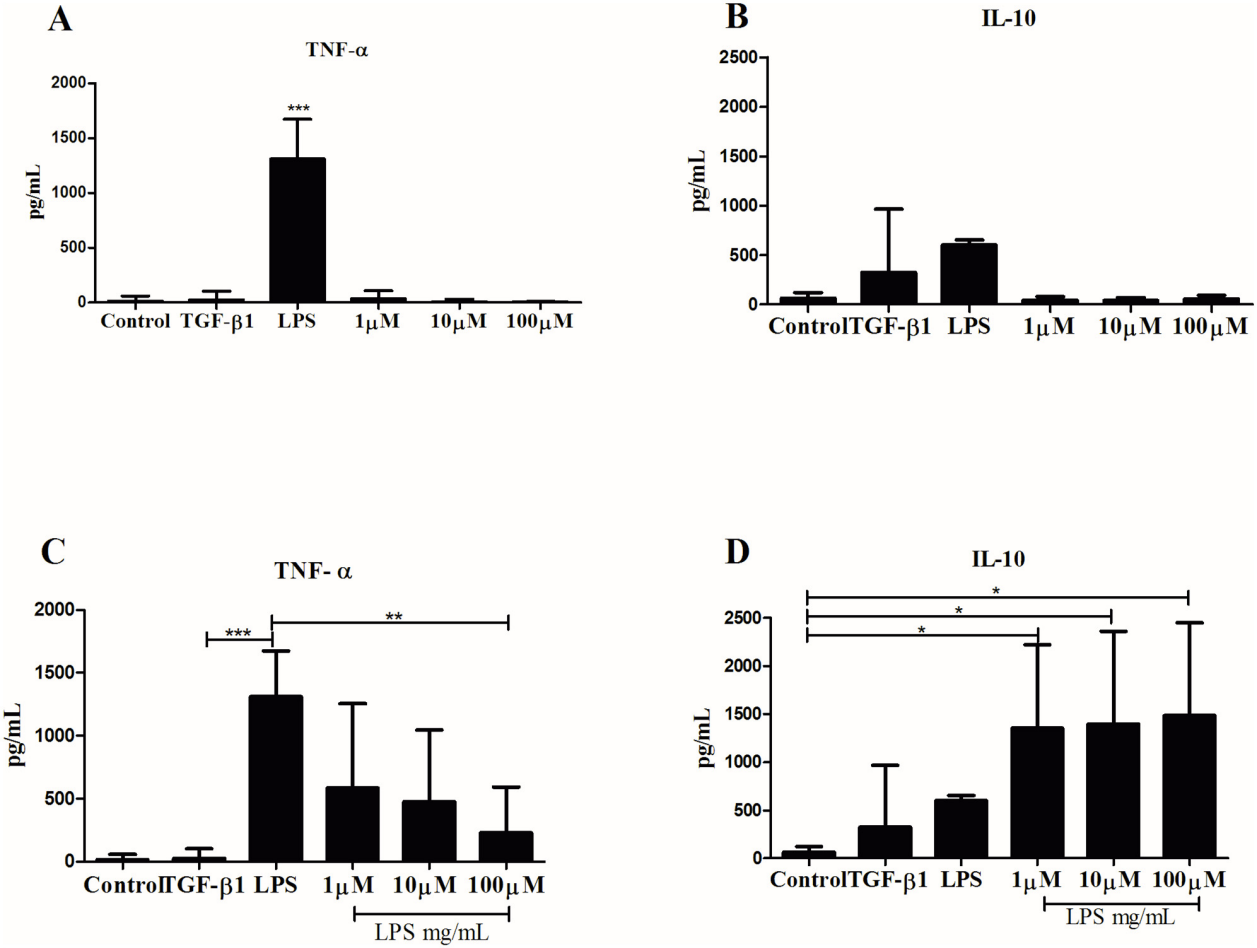
Analysis of cytokines production by PBMC after stimulation for 24 hours. Analysis of TNF-α (A) and IL-10 (B) cytokines released in the absence of inflammatory stimulus. Analysis of TNF-α (C) and IL-10 (D) cytokines released after PBMC stimulation with LPS. PBMC pretreated with the pm26TGF-β1 peptide at 100 μM was able to decrease TNF-α production (P < 0.001). PBMC pretreated with the peptide followed by stimulation with LPS resulted in a significant increase in IL-10 production when compared to control (P < 0.05).

**Fig 6:**
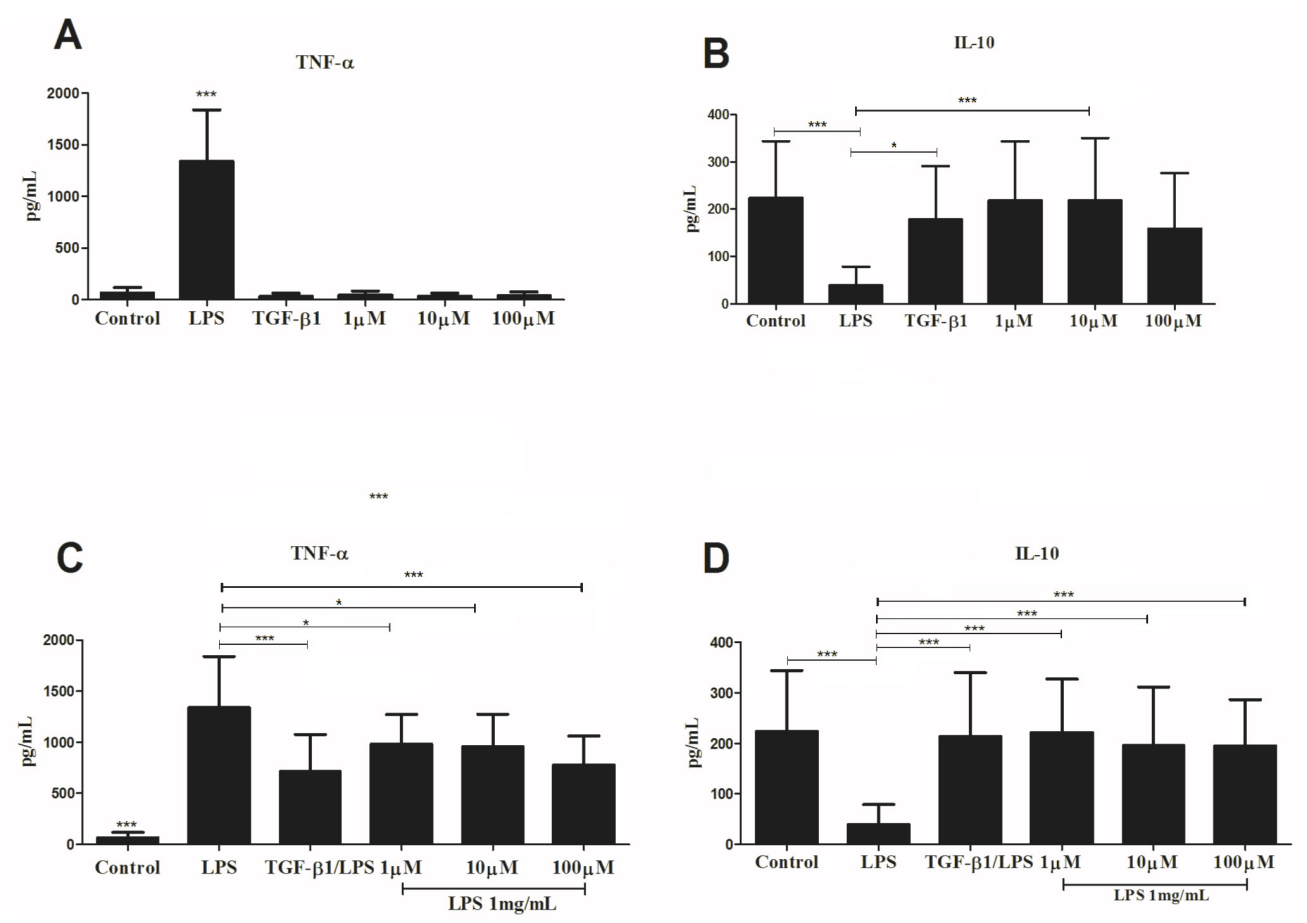
Analysis of cytokines production by PBMC after stimulation for 48 hours. Analysis of TNF-α (A) and IL-10 (B) cytokines released in the absence of inflammatory stimulus. Analysis of TNF-α (C) and IL-10 (D) cytokines released after PBMC stimulation with LPS. TNF-α production was decreased after PBMC pretreated with the pm26TGF-β1 peptide (1 μM and 10 μM; P < 0.05) and 100 μM (P < 0.0001). PBMC pretreated with the peptide followed by stimulation with LPS showed no difference compared to LPS/TGF-β1.

To verify whether the pm26TGF-β1 peptide has the same ability of TGF-β1 to modulate an immune response, we have stimulated PBMCs, and measured TNF-α and IL-10 production. The pm26TGF-β1 peptide was not able to induce TNF-α (Fig 5A) or IL-10 (Fig 5B) production in the absence of inflammatory stimulus. PBMC pretreated with the pm26TGF-β1 synthetic peptide followed by LPS stimulation for 24 hours presented significant decrease in TNF-α production (P < 0.001) when compared to LPS-treated cells (Fig 5C). Interestingly, all concentrations of peptide used for cell stimulation resulted in a significant increase in IL-10 production when compared to controls (P < 0.05) (Fig 5D). Furthermore, the efficiency of pm26TGF-β1 in down-modulating TNF-α and up-regulating IL-10 was confirmed after 48h-stimulus of PBMCs, as performed in 24 hours. Similarly, the synthetic peptide presented no action on TNF-α (Fig 6A) and IL-10 (Fig 6B) production in the absence of inflammatory stimulus. Contrarily, the LPS stimulus induced a significant inflammatory response, as expected. The combination between LPS and the recombinant TGF-β1 or the pm26TGF-β1 (1 μM and 10 μM, P < 0.05; and 100 μM, P < 0.0001) reduced significantly the TNF-α production (Fig 6C) and increased IL-10 production (Fig 6D). Briefly, the pm26TGF-β1 peptide showed a similar behavior of TGF-β1, modulating both inflammatory and anti-inflammatory responses.

### Induction of regulatory T cells

**Fig 7:**
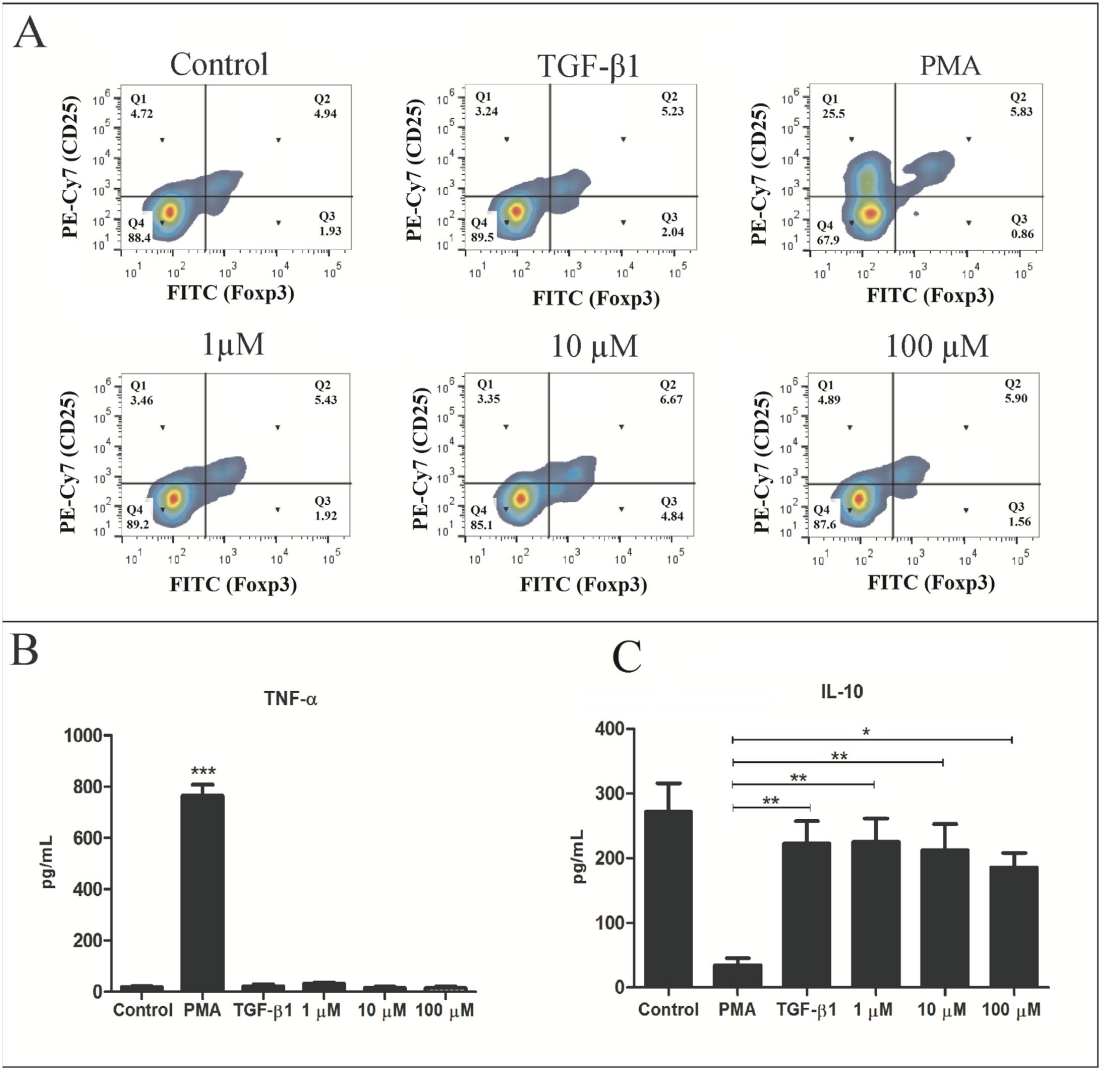
Analysis of cytokines production and expression of CD4^+^ CD25^+^ Foxp3^+^ cells. (A) Treg cells percentage after pm26TGF-β1 peptide stimulation in different concentrations and controls. Analysis of TNF-α (B) and IL-10 (C) cytokines released after PBMC stimulation with pm26TGF-β1 peptide.

To verify if the peptide could induce Treg cells, we have compared the percentage obtained by PBMC stimulated with TGF-β1 and pm26TGF-β1 peptide. Tregs percentage of untreated PBMC was 4.94%, while after stimulus with recombinant TGF-β1 and PMA there was an increased Tregs percentage of 5.23% and 5.83%, respectively (Fig 7A). The pm26TGF-β1 peptide stimulus at 1 μM, 10 μM and 100 μM presented Tregs percentage of 5.43%, 6.67% and 5.90%, respectively. Thus, pm26TGF-β1 at 100 μM induced a 35% increase of Tregs compared to controls, while TGF-β induced only 5.8%, showing the pm26TGF-β1 peptide ability to induce Treg differentiation. Cytokines quantification present in the cell culture supernatant reinforces that the pm26TGF-β1 peptide presented no action in the TNF-α (Fig 7B) and IL-10 (Fig 7C) in the absence of inflammatory stimulus. Our data support the pm26TGF-β1 peptide role in inducing Treg cells, similar to the TGF-β1 function, further evidencing its mimicking ability.

### Analysis of leucocyte rolling *in vivo*

**Fig 8:**
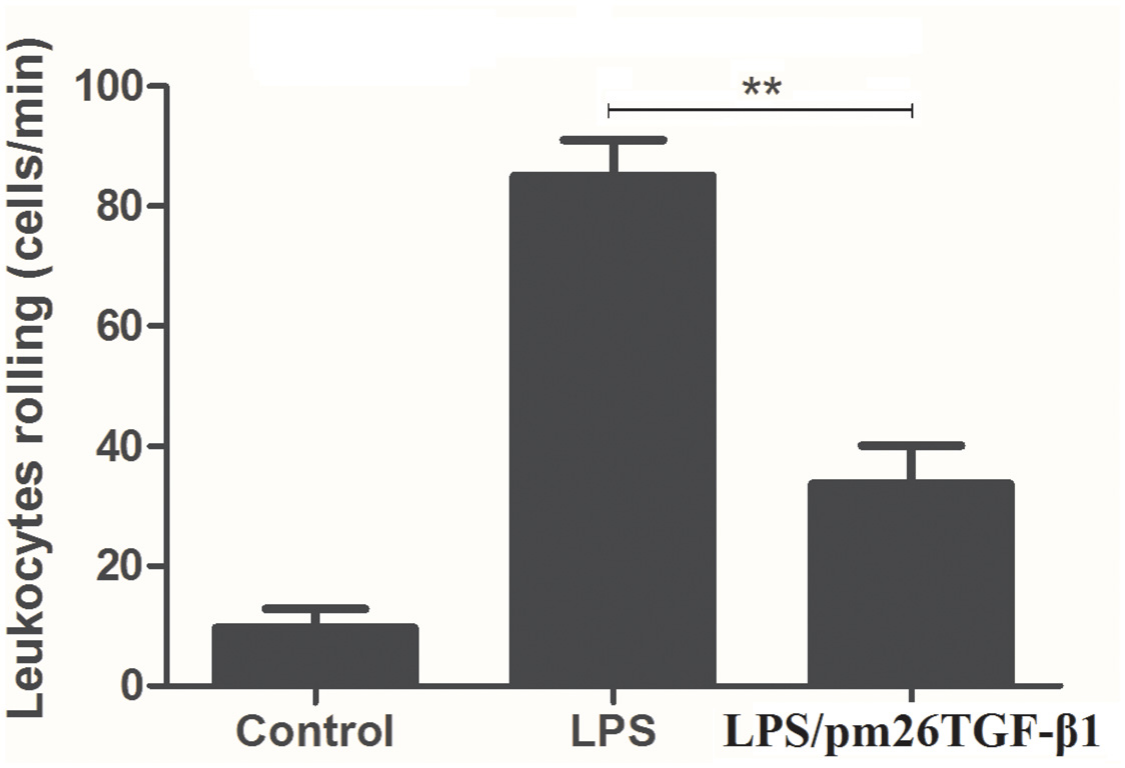
Analysis of leucocyte rolling *in vivo*. The pretreatment with LPS followed by treatment with pm26TGF-β1 peptide reduced leukocytes rolling in mice after 30 min of treatment (P < 0.001).

The leukocyte rolling and adhesion cascade is an important event during the inflammatory response, which was used to demonstrate whether the pm26TGF-β1 peptide was able to disturb this process as an anti-inflammatory response. Leukocytes’ rolling was decreased (60.4%; P < 0.001) in mice pretreated with LPS followed by pm26TGF-β1 peptide administration compared to the control mice treated with LPS (Fig 8). We demonstrated through an *in vivo* assay that the pm26TGF-β1 peptide was able to affect the leukocyte rolling process, consequently reducing the inflammatory response.

### Peritonitis assay

**Fig 9:**
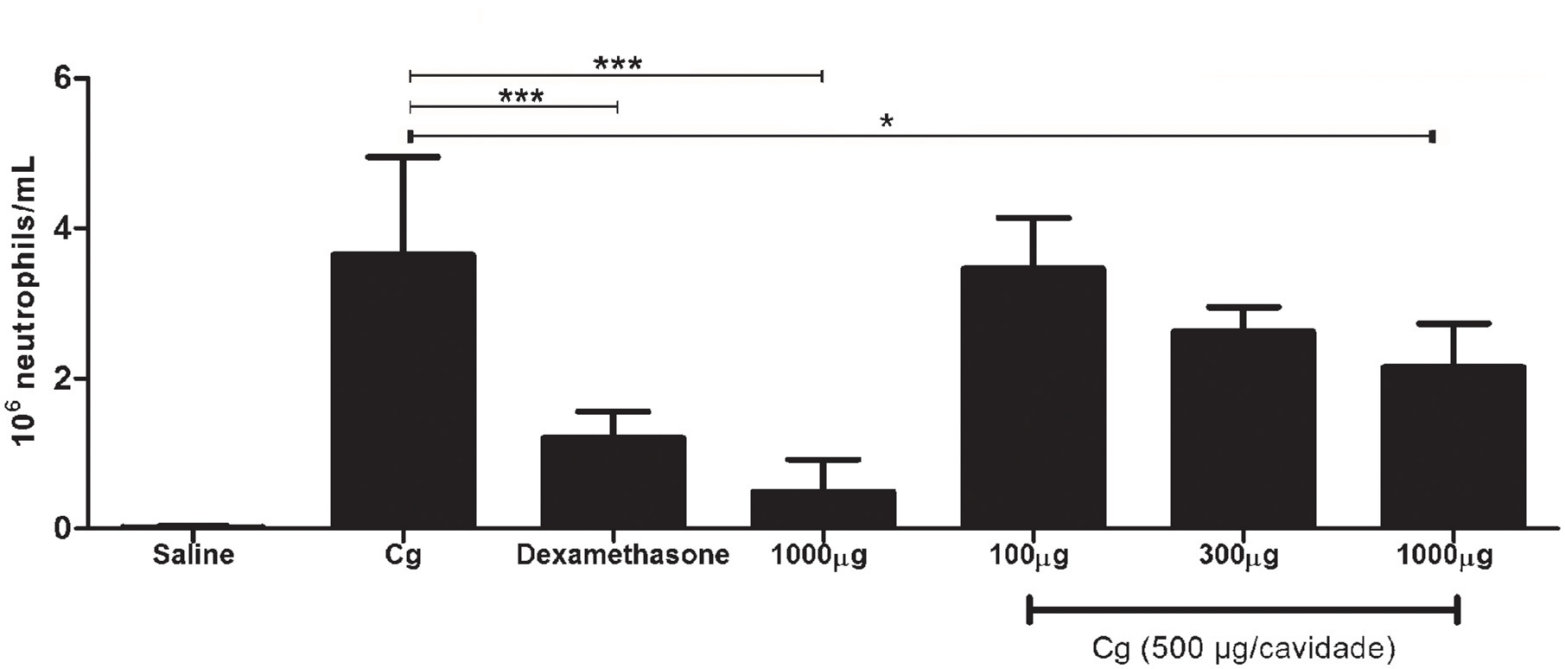
Neutrophil migration analysis in vivo. The pretreatment with the pm26TGF-β1 peptide reduced neutrophils migration into peritoneal cavity (P < 0.01). Dexamethasone was used to suppress the immune response induced by carrageenan, and higher concentration of pm26TGF-β1 peptide without carrageenan (1000 μg) was used to confirm that the peptide is not able to induce a nonspecific response.

A second inflammatory model was used to further demonstrate whether the peptide could also modulate the neutrophil migration in peritonitis induction model. Stimulation in C57BL/6 mice with the pm26TGF-β1 peptide efficiently decreased neutrophils migration into intraperitoneal fluid when compared to the control mice treated with carrageenan (P < 0.01) (Fig 9). Dexamethasone was used to suppress the immune response, and reduced about 66% of total neutrophil migration, while the peptide reduced about 40%, reinforcing that the pm26TGF-β1 peptide presents a potential anti-inflammatory action.

### Pm26TGF-β1 mimics TGF- β1

**Fig 10:**
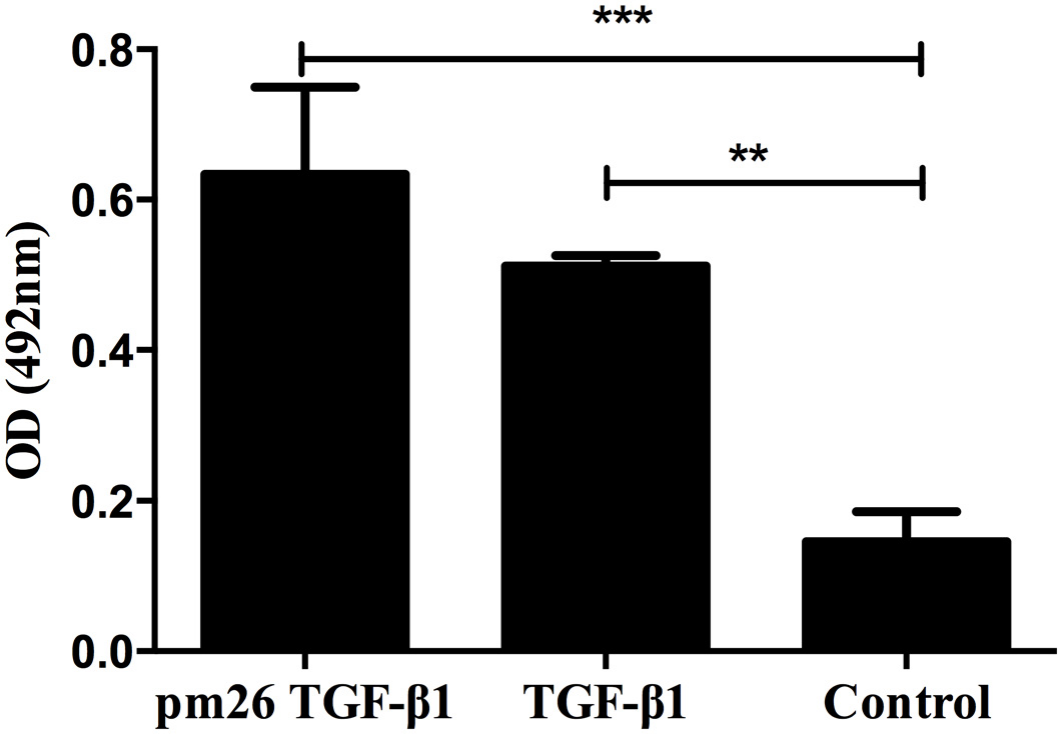
Anti-TGF-β1 antibody recognizes pm26TGF-β1. The commercial anti-TGF-β1 antibody recognized the pm26TGF-β1 peptide, bound in PBMC surface, confirming that the TβRII has equally recognized both TGF-β1 and pm26TGF-β1 peptide.

In order to demonstrate whether the pm26TGF-β1 peptide is truly mimicking the TGF-β1, we first performed a competitive elution of the peptide library against PBMC cells with recombinant TGF-β1. Secondly, we have shown that TNF-α was down regulated and IL-10 up regulated after PBMC stimuli (Figs 5 and 6). The third evidence was provided by 3D prediction analyses, including the peptide alignment to TGF-β1 structure (Fig 2) and interaction with TβRII (Fig 3). The fourth evidence came from *in vitro* (Figs 5–7) and *in vivo* tests (Figs 8 and 9), which confirmed that the pm26TGF-β1 was able to modulate the inflammatory response, as expected when TβRII is activated. Finally, we have demonstrated that the pm26TGF-β1 peptide is really mimicking TGF-β1 by direct proof against a TGF-β1 monoclonal antibody, which was performed in PBMC cells, confirming that the TβRII has equally recognized both TGF-β1 and the pm26TGF-β1 peptide (Fig 10).

## Discussion

The great demand for new drugs with low toxicity to control excessive inflammation in inflammatory diseases is becoming one of the major targets of the pharmaceutical industry. The development of drugs associated with the TGF-β1 pathway to control autoimmune diseases is desired due to the expected dual function of controlling self-reactive T cells and the inflammatory response. Considering that the structure of the receptor-ligand pairing in the TGF-β superfamily presents different affinities at their interface [32], we hypothesized that peptides that mimic the binding domain may behave and function differentially according to different insertions, deletions, and disulfide bonds in the binding domain. Therefore, our study aimed to search for new bioactive peptides through PhD technology. Our strategy has resulted in a successful TGF-β1-like peptide with similar or better potency than the native molecule, which will be discussed herein.

Depletion of CD4^+^CD25^+^T cells from wild-type mice has led to the spontaneous development of several inflammatory diseases, and the reconstitution of CD4^+^CD25^+^T cells in these animals has prevented the development of these diseases [33]. The nTreg and iTreg cells are phenotypically indistinguishable, and interestingly, the iTreg repertoire is more specific for tissue and foreign antigens and more functionally active at inflammatory sites [34], which is the scope of this investigation.

The association of random peptide libraries expressed on filamentous phage surface with effective *in vitro* and *in vivo* selection strategies has been very successful in the identification of novel peptide ligands in many different systems and cell types [35–39]. Using a competitive strategy for peptides elution, we have successfully selected a TGF-β1-like peptide that recognizes the TβRII, which was fully demonstrated by *in vitro* and *in vivo* assays, and by structural analysis. The pm26TGF-β1 peptide was mapped into the TGF-β1:TβRII activation domain, characterized by the interaction between the Phe^30^, Asp^32^, Ser^49^, Ile^50^, Ser^52^, Ile^53^ and Glu^119^ residues at the TβRII and the Arg^25^, His^34^, Tyr^91^, Gly^93^ and Arg^94^ residues at the TGF-β1 molecule [32]. It has been shown that the residues Arg^25^ and Arg^94^ present in the binding site of TGF-β1 are crucial for hydrogen bonds formation with its receptor [32],[40]. These two residues are responsible for over 30% of total binding energy, which results in a high-affinity interaction between isoforms of this molecule and TβRII receptor [41]. The Ser^49^ and Glu^119^ residues present in TβRII interact with the Glu and Lys present in the pm26TGF-β1 peptide, which interestingly is the same binding domain recognized by the TGF-β1. Furthermore, the presence of Arg residue in the pm26TGF-β1 peptide, similarly to what occurs in TGF-β1, forms hydrogen bonds that stabilize the peptide/TβRII binding [32]. Interestingly, the other tested peptide, pm1TGF-β1, despite not sharing residues with TGF-β1, was able to significantly interact with receptors present on PBMC, with similar function shown for the pm26TGF-β1 peptide. This result demonstrates that specific structure conformations can be achieved by mimetic peptides without sharing any residue of the binding domain, and reaffirming the notion that mimetic peptides may represent an alternative for bioactive molecules’ development.

The pm26TGF-β1 peptide was able to decreases TNF-α and IL-10 release only in an inflammatory microenvironment. PBMC stimuli was performed with LPS and PMA, which are agonists of toll-like receptor 4 [42] and protein kinase C [43], respectively, both associated with NF-κB activation [44] that triggers TNF-α [45], IL-1β and COX2 production [46], the active transcription of some inflammatory genes [42], and production of reactive oxygen species (ROS) [47]. Importantly, a balanced immune response is preferred, and IL-10 increase is also essential for the immune response modulation, especially when associated with TNF-α decrease, which may prevent excessive inflammation and tissue damage [48], which cannot be achieved by simply inhibiting a unique cytokine. This explains the reason that led us to select novel TGF-β1 mimetic peptides that could modulate the inflammatory response by activating iTregs.

The pm26TGF-β1 peptide has efficiently induced the regulatory T cells phenotype (CD4^+^CD25^+^Foxp3^+^) in 24 hours after PBMC stimulation, which is considered a short time for cellular induction [49]. PMA was used as a positive control, which is considered a standard stimulus for peripherally induced iTregs [50]. The overall Tregs percentage in PBMC of healthy individuals is generally small [51] and its regulatory potential oscillate from 2% to 3% of cells with high CD25^+^ (CD25^hi^) expression [52]. In our study, the iTregs percentage in health individuals was approximately 5%, including CD25^hi^ and CD25^low^ cells, from which approximately 2% to 3% was CD25^hi^, suggesting an immunomodulatory role capable of producing cytokines with regulatory action. Furthermore, compared to TGF-β1, Foxp3 was higher detected after stimulation with pm26TGF-β1 peptide.

Development and progression of autoimmune diseases are related to changes in number and function of Tregs [53, 54]. These cells can stop to play a suppressive role on T cells of patients with autoimmune disorders, as multiple sclerosis [55] and type I diabetes [56]. In patients with rheumatoid arthritis, the number of Tregs can be normal; however, these cells may not be effective in decreasing TNF-α release [57]. The greatest achievement of this work was the development of a peptide that did not induce significant changes in the number of differentiated Tregs in human PBMC. Furthermore, the peptide could down-regulate TNF-α and up-regulate IL-10 production during inflammatory stimuli, a profile that is required in the treatment of inflammatory diseases [58].

In rheumatoid arthritis, for example, TGF-β1 can suppress pro-inflammatory cytokines secretion, such as INF-γ and TNF-α, due to Treg cells induction in the periphery [22]. Failure in the TGF-β1 receptor in a mouse model resulted in an autoimmune event that led to injuries and death, demonstrating its importance in the prevention and disease treatment [59]. Treatment with this cytokine in early diabetes development is effective in the inhibition of self-reactive T cells in the pancreas, preventing the disease progression [60]. In addition, the IL-10 increase after stimulations with pm26TGF-β1 peptide, may also be associated with the immune suppression [61] mediated by Tregs in experimental models [62] and human PBMC [63].

Increase in TNF-α production is directly related to the PMN increase [64, 65]. Pro-inflammatory cytokines activate the endothelium and promote E-selectin expression, which is responsible for inducing slow leukocytes rolling due to the integrins’ activation [66, 67]. Although TNF-α has not been measured in the *in vivo* assays, we hypothesized that the decrease in neutrophils infiltration and leukocytes rolling might be a consequence of the TNF-α inhibition, as evidenced in all *in vitro* assays. The reason for this might be that TNF-α is responsible for leukocytes adhesion to the endothelium, and the increased expression of E-selectin and ICAM-1 activates inflammation, resulting in leukocytes and neutrophils increase [68].

*In vivo* anti-inflammatory response of the pm26TGF-β1 peptide in carrageenan-induced peritonitis model demonstrated that this peptide might have direct inhibitory effects on neutrophils migration. Carrageenan is a β-glucan molecule used to induce inflammation in a variety of experimental models by inducing rapid release of pro-inflammatory cytokines, such as TNF-α, and accentuated neutrophil migration [69]. TNF-α and IL-1β secretion is promoted early after stimulus, and after 2 to 6 hours, neutrophils infiltration can be observed at the intraperitoneal cavity [70] with increased ICAM-1 expression [71], which reinforces the pivotal role of neutrophils in combating inflammation [72]. It has been demonstrated that TGF-β inhibits both basal TNF- and IL-1-stimulated expression of E-selectin [73] and neutrophils transmigration through endothelial monolayers [74]. TGF-β1 treatment can also inhibit NFκB-mediated E-selectin gene expression quickly (about 30 min) [75], which is enough to prevent an increase in its expression induced by carrageenan in our work. E-selectin is an important molecule involved in leukocyte rolling and neutrophil migration [66, 76], and its decrease could explain the fast reduction in both events, as demonstrated by in vivo experiments after pretreatment with the pm26TGF-β1. Additionally, the capacity of the peptide in decreasing TNF-α and increasing IL-10 may also reduce leukocyte rolling and neutrophil migration. Although these findings suggest a potential use of the pm26TGF-β1 peptide in the inflammatory modulation, the exact mechanism must be further explored in future studies.
